# Supplementary material for: Parenchymal Sparing Surgery for Lung Cancer: Focus on Pulmonary Artery Reconstruction
Source: Cancers (Basel). 2022 Sep 30;14(19):4782. doi: 10.3390/cancers14194782 (PMC9563968; doi:10.3390/cancers14194782)
Supplement: Supplementary file 1 [file cancers-14-04782-s001.zip › cancers-1887852-supplementary.pdf]

cancers

MDPI

Supplementary

Parenchymal Sparing Surgery for Lung Cancer: Focus on Pulmonary Artery Reconstruction

Table S1. Results of studies reporting PA reconstruction for lung cancer.

Author (y)	Pts (n)	Tangential Suture (n)	End-to-End Anastomosis (n)	Patch (n)	Conduit (n)	Morbidity (%)	Mortality (%)	Survival (5 y) (%)
Rendina et al,[27] 1999	52	0	15	34	3	13.4	0	38.3
Shrager et al,[41] 2000	33	19	3	11	0	45.0	0	46.7 (4 y)
Lausberg et al,[14] 2005	67	0	39	28	0	34	1.5	42.9
Nagayasu et al,[46] 2006	29	0	17	12	0	27.6	17.2	24.2
Cerfolio et al,[24] 2007	42	31	4	7	0	26.0	2.3	60.0
Alifano et al,[45] 2009	93	88	3	2	0	29.0	5.4	39.4
Venuta et al,[28] 2009	105	0	47	55	3	28.5	0.95	44.0
Barthet et al,[31] 2013	32	0	20	2	10	40*	0	66.7
D'Andrilli et al,[30] 2014	9	0	0	0	9	33.3	0	NA (median surv time 38 months)
D'Andrilli et al,[51] 2018	24	0	0	4	20	29.1	0	69.9
Watanabe et al,[60] 2022	130	56	32	26	16	57.7	1.5	49.2
Yang et al,[54] 2022	139	NA	NA	NA	NA	28.1	0	73.5 (3 y)
Hattori et al,[53] 2022	17	4	8	1	4	58.8	2.3	29.4 (3 y)

* Considering only 10 patients who received PA replacement with cryopreserved PA and thoracic aorta grafts from multiorgan donors.

Table S2. Results of studies reporting complication rates after PA reconstruction.

Author (y)	Overall Complications Rate (%)	PA Trombosis (n/%)	Bleeding (n/%)	Cardiac (n/%)	Chylothorax (n/%)	Pneumonia (n/%)	Other (n/%)
Rendina et al,[27] 1999	13.4	1/1.9	0/0	1/1.9	0/0	0/0	5/9.6
Shrager et al,[41] 2000	45.1	0/0	0/0	4/8.9	0/0	2/4.4	12/31.1
Lausberg et al,[14] 2005	34	0/0	0/0	0/0	0/0	NA	23/34
Nagayasu et al,[46] 2006	27.6	1/3.4	0/0	2/6.8	0/0	0/0	17.2
Cerfolio et al,[24] 2007	38.1	0/0	0/0	6/14.3	0/0	1/2.4	9/21.4
Alifano et al,[45] 2009	29.0	0/0	0/0	8/8.6	0/0	12/12.9	14/14.0
Venuta et al,[28] 2009	28.5	1/0.9	1/0.9	5/4.8	0/0	0/0	23/21.9
Barthet et al,[31] 2013	40*	1/10	0/0	0/0	0/0	0/0	3/30
D'Andrilli et al,[30] 2014	33.3	0/0	0/0	1/11.1	1/11.1	0/0	1/11.1
D'Andrilli et al,[51] 2018	7/29.1	0/0	1/4.2	4/16.7	1/4.2	0/0	1/4.2
Watanabe et al,[60] 2022	57.7	2/1.5	2/1.5	27/20.8	4/3.1	7/5.4	33/25.4
Yang et al,[54] 2022	28.1	0/0	0/0	1/0.7	1/0.7	14/10.3	18/12.9
Hattori et al,[53] 2022	58.8	1/5.9	0/0	3/17.6	2/11.8	0/0	4/23.5

* Considering only 10 patients who received PA replacement with cryopreserved PA and thoracic aorta grafts from multiorgan donors.
